# Machine Learning-Based Assessment of Cognitive Impairment Using Time-Resolved Near-Infrared Spectroscopy and Basic Blood Test

**DOI:** 10.3389/fneur.2021.624063

**Published:** 2022-01-27

**Authors:** Katsunori Oyama, Kaoru Sakatani

**Affiliations:** ^1^Department of Computer Science, Nihon University, Koriyama, Japan; ^2^Department of Human and Engineered Environmental Studies, Graduate School of Frontier Sciences, The University of Tokyo, Tokyo, Japan

**Keywords:** mini-mental state examination, time-resolved near-infrared spectroscopy, basic blood test, linear model, deep neural network for regression

## Abstract

We have demonstrated that machine learning allows us to predict cognitive function in aged people using near-infrared spectroscopy (NIRS) data or basic blood test data. However, the following points are not yet clear: first, whether there are differences in prediction accuracy between NIRS and blood test data; second, whether there are differences in prediction accuracy for cognitive function in linear models and non-linear models; and third, whether there are changes in prediction accuracy when both NIRS and blood test data are added to the input layer. We used a linear regression model (LR) for the linear model and random forest (RF) and deep neural network (DNN) for the non-linear model. We studied 250 participants (mean age = 73.3 ± 12.6 years) and assessed cognitive function using the Mini Mental State Examination (MMSE) (mean MMSE scores = 22.9 ± 6.1). We used time-resolved NIRS (TNIRS) to measure absolute concentrations of hemoglobin and optical pathlength at rest in the bilateral prefrontal cortices. A basic blood test was performed on the same day. We compared predicted MMSE scores and grand truth MMSE scores; prediction accuracies were evaluated using mean absolute error (MAE) and mean absolute percentage error (MAPE). We found that (1) the DNN-based prediction using TNIRS data exhibited lower MAE and MAPE compared with those using blood test data, (2) the difference in MAPE between TNIRS and blood test data was only 0.3%, (3) adding TNIRS data to the blood test data of the input layer only improved MAPE by 1.0% compared to the use of blood test data alone, whereas the use of the blood test data alone exhibited the prediction accuracy with 81.8% sensitivity and 91.3% specificity (*N* = 202, repeated five-fold cross validation). Given these findings and the benefits of using blood test data (low cost and large-scale screening possible), we concluded that the DNN model using blood test data is still the most suitable for mass screening.

## Introduction

According to the World Alzheimer Report, the population of Alzheimer's disease (AD) patients is predicted to grow to over 100 million by 2050 (1). However, at present, there are no drugs that can cure advance dementia, and therefore, delaying the onset of dementia has received great attention (2). To this end, screening tests for cognitive dysfunction play a crucial role in prevention of dementia. Currently, the Mini Mental State Examination (MMSE) is the most commonly used scale in cognitive function evaluation (3). The MMSE is sensitive and cost-effective screening test; however, it is a subjective examination, and does not allow clinicians to examine a large number of patients in a short time since it is carried out one by one between the clinicians and the patients. In addition, the MMSE is difficult to perform on patients with neurological disorders such as visual and hearing impairment.

Recently, we have developed a deep learning (DL)-based screening test of cognitive impairment which used a basic blood test data for health examinations (4). The DL-based screening test was developed based on the relation between cognitive function and systemic metabolic disorders in aged people. That is, atherosclerosis induced by lifestyle-related diseases could cause vascular cognitive impairment (VCI) through cerebral ischemia, which plays an important role in dementia onset of not only vascular dementia but also Alzheimer's disease (AD) in the elderly (5). To analyze the complex non-linear relationships between systemic metabolic disorders and cognitive function, we used a deep neural network (DNN). Employing DNNs, we analyzed the relationship between 23 blood test items and participant's ages (input) and the ground truth MMSE scores in the participants (output). Validation of the DNN model showed higher prediction accuracy than the results of linear regression models; we validated the prediction accuracy in the training data (by a leave-one-out cross-validation) and in the test data that were not used for training the DNN model.

In contrast to basic blood test for health examination, a time-resolved near infrared spectroscopy (TNIRS) allows direct measurements of cerebral hemodynamics and optical characteristics such as optical pathlength which reflect brain function and structural changes of the brain (6). Near infrared spectroscopy is an optical method which allows for measurement of hemoglobin (Hb) concentrations in cerebral blood vessels (7). Recently, we used a TNIRS, which measures Hb concentrations at rest, to assess cognitive function in aged people, e.g., persons 60 years and older (8). We found that concentrations of oxyhemoglobin and oxygen saturation (SO_2_) in the prefrontal cortex (PFC) at rest correlated with cognitive function assessed by the MMSE in aged people (9). Therefore, using the same DNN as above, we predicted cognitive functions by putting the TNIRS parameters into the input layer of the DNN (10) and observed prediction accuracies for the DNN model. However, it is unclear which is more accurate in predicting the MMSE score, with TNIRS parameters that reflect cerebral hemodynamics and general blood test data that reflect systemic metabolic status. In addition, we do not know whether there are changes in prediction accuracy when both TNIRS and blood test data are added to the input layer. Moreover, it is not yet clear whether there are substantial differences in prediction accuracy for cognitive function between DNN and the baseline models including the linear regression (LR) model and random forest (RF) as one of the non-linear models.

In order to address these issues, we performed the following analysis: first, we compared the prediction accuracies of the MMSE score between TNIRS parameters and blood test data. Second, we evaluated the effect of combination of TNIRS and blood test data on the prediction accuracy. Finally, we compared the prediction accuracies of LR, RF and DNN models. A LR model for the linear model and RF and DNN for the non-linear model are used in the analysis. This study used a group of participants who performed both TNIRS measurements and blood tests at the same time.

## Methods

### Cognitive Screening Test Using the Mini-Mental State Examination

We studied 250 participants [115 males, 135 females; age 73.3 ± 12.6 (mean ± SD) range 27–100 (minimum – maximum); 188 participants aged ≥ 65 years, 62 participants aged <65 years] who exhibited a variety of cognitive functions ranging from normal to dementia. All participants had visited Southern Touhoku Kasuga Rehabilitation Hospital (Sukagawa City, Japan) due to various symptoms, including forgetfulness. The participants provided written informed consent as required by the Human Subjects Committee of the Rehabilitation Hospital; when the participant had difficulty understanding the concept of informed consent due to cognitive dysfunction, the participant's family provided consent.

Initially, we evaluated each participant's cognitive function using the MMSE, which is an effective screening tool that has been used to systematically assess mental status (11) using cut-off values of ≥ 24 for normal and <24 for risk of cognitive impairment. The mean MMSE score was 22.9 ± 6.1 (mean ± SD) range 4–30 (minimum – maximum); 135 cases were normal, whereas the other 115 cases showed risk of cognitive impairment. These MMSE scores were used to calculate residual error from the estimated scores using machine learning techniques with the input of age, blood test data, and TNIRS data as discussed in the following sections.

### Time-Resolved Near-Infrared Spectroscopy Measurement and Blood Test

Hb concentrations at rest in the bilateral PFC were measured using a two-channel TNIRS system (TRS-21, Hamamatsu Photonics K.K., Hamamatsu, Japan). Details of this system have been described previously (12). Briefly, it consists of three pulsed laser diodes with different wavelengths (760, 790, and 830 nm) having a pulse duration of 100 ps at a repetition frequency of 5 MHz, a photomultiplier tube (H6279-MOD, Hamamatsu Photonics K.K., Japan), and a circuit for time-resolved measurement based on the time-correlated single photon counting technique. The concentrations of Hb were expressed in μM. In addition, we performed a blood test that included a complete blood count and a basic metabolic panel at the time of the experiment for all the participants. The measurement data are shown in Table 1. The total number of feature values from TNIRS parameters was 14 (i.e., 7 items with 2 channels as the TNIRS probes were set symmetrically on the forehead, with a flexible fixation pad at the left frontal pole and right frontal pole). The numbers of feature values from the complete blood count and general biochemical examinations were 8 and 15, respectively.

**Table 1:** TNIRS parameters and blood test data.

**TNIRS parameters**	**Complete blood count**	**General biochemical examination**	
Oxy-Hb (L, R)	WBC count	Total Protein	BUN
Deoxy-Hb (L, R)	RBC count	Albumin	Creatinine
Total-Hb (L, R)	Hemoglobin	A/G ratio	Uric Acid
SO_2_ (L, R)	Hematocrit	AST (GOT)	Glucose
OP1: 760 nm LED (L, R)	MCV	ALT (GPT)	Na
OP2: 790 nm LED (L, R)	MCH	r-GTP	K
OP3: 830 nm LED (L, R)	MCHC	Total cholesterol	Cl
	Platelet count	Triglyceride	

All the measured data are summarized in Table 2. *MMSE score* is the response variable for all machine learning methods in this study. Using the cut-off values of MMSE score, the Normal group is the participants whose MMSE score of 24 and above, whereas the Impaired group is the participants whose MMSE score under 24. Results from Spearman's correlation analysis showed that *MMSE score* was significantly correlated (*r* > 0.2) with *Age, SO*_2_, *RBC, Alb, Na* and *Cl*. It is noteworthy that the optical path lengths (i.e., *OP1, OP2*, and *OP3*) were higher in the Impaired group (i.e., participants whose MMSE score was below 24 showing risk of cognitive impairment), and *OP2* and *OP3* in the right PFC were significantly different between the Normal and Impaired groups. These variables are important for achievement of higher prediction accuracy in the learning models.

**Table 2:** Measured data.

**Data type**	**Item**	**All patients (*N* = 250)**	**Correlation to MMSE score**	**Normal (*N* = 135)**	**Impaired (N=115)**	**Difference (normal vs. impaired)**
Response variable	MMSE score	22.9 ± 6.1	–	27.4 ± 2.1	17.5 ± 4.7	***p*** **<** **0.001**
Demographics	Age	73.3 ± 12.6	**−0.42**	68.4 ± 13.1	79.1 ± 9.0	***p*** **<** **0.001**
	Sex	M:115 F:135	–	M:67 F:68	M:48 F:67	
TNIRS	OP1 _R_	19.1 ± 1.7	−0.07	19.0 ± 1.7	19.3 ± 1.8	*p* = 0.33
	OP2 _R_	19.5 ± 1.8	−0.12	19.4 ± 1.7	19.8 ± 1.9	***p*** **=** **0.09**
	OP3 _R_	18.4 ± 1.7	−0.12	18.2 ± 1.6	18.6 ± 1.8	***p*** **=** **0.08**
	HbO_2__R_	38.2 ± 8.3	0.16	39.2 ± 8.3	37.0 ± 8.0	***p*** **=** **0.04**
	Hb _R_	18.8 ± 3.9	−0.07	18.5 ± 3.9	19.2 ± 3.8	*p* = 0.15
	tHb _R_	57.0 ± 11.3	0.09	57.7 ± 11.6	56.2 ± 10.8	*p* = 0.29
	SO_2__R_	66.8 ± 3.7	**0.30**	67.8 ± 3.3	65.6 ± 3.8	***p*** **<** **0.001**
	OP1 _L_	19.0 ± 1.7	0.01	19.0 ± 1.7	19.0 ± 1.7	*p* = 0.92
	OP2 _L_	19.3 ± 1.8	−0.04	19.2 ± 1.7	19.4 ± 1.8	*p* = 0.47
	OP3 _L_	18.0 ± 1.6	−0.04	17.9 ± 1.6	18.0 ± 1.7	*p* = 0.54
	HbO_2L_	36.8 ± 8.0	0.09	37.6 ± 8.1	35.9 ± 7.9	*p* = 0.10
	Hb _L_	18.4 ± 3.9	−0.15	17.9 ± 4.0	18.9 ± 3.7	***p*** **=** **0.03**
	tHb _L_	55.2 ± 10.9	0.01	55.4 ± 11.3	54.9 ± 10.5	*p* = 0.68
	SO_2L_	66.6 ± 4.0	**0.30**	67.7 ± 3.6	65.3 ± 4.1	***p*** **<** **0.001**
Blood test	WBC	5,865.2 ± 1,780.9	0.06	6,031.1 ± 1,837.2	5,670.4 ± 1,699.7	*p* = 0.11
	RBC	420.0 ± 58.2	**0.22**	432.6 ± 56.0	405.3 ± 57.4	***p*** **<** **0.001**
	Hb	13.2 ± 7.8	0.05	13.9 ± 10.5	12.3 ± 1.7	*p* = 0.11
	Ht	38.9 ± 4.8	0.18	39.8 ± 4.7	37.9 ± 4.7	***p*** **=** **0.001**
	MCV	92.6 ± 7.3	−0.10	91.8 ± 8.7	93.5 ± 4.9	***p*** **=** **0.07**
	MCH	30.2 ± 1.9	−0.09	30.1 ± 1.9	30.4 ± 1.9	*p* = 0.22
	MCHC	32.6 ± 1.2	0.06	32.6 ± 1.3	32.5 ± 0.9	*p* = 0.36
	PLT	23.8 ± 6.7	0.19	25.1 ± 7.0	22.3 ± 6.1	***p*** **=** **0.001**
	TP	7.1 ± 3.6	0.01	7.0 ± 0.6	7.2 ± 5.3	*p* = 0.64
	Alb	3.9 ± 0.4	0.27	4.0 ± 0.4	3.7 ± 0.5	***p*** **<** **0.001**
	A/G	1.3 ± 0.3	0.14	1.3 ± 0.3	1.3 ± 0.3	***p*** **=** **0.02**
	AST-GOT	22.0 ± 9.2	0.05	22.2 ± 9.9	21.9 ± 8.2	*p* = 0.84
	ALT-GPT	20.6 ± 17.7	0.13	22.5 ± 21.1	18.3 ± 12.3	***p*** **=** **0.06**
	r-GT	28.1 ± 25.1	0.18	31.3 ± 25.3	24.4 ± 24.5	***p*** **=** **0.04**
	T-cho	177.5 ± 38.8	0.12	180.4 ± 35.9	174.1 ± 41.6	*p* = 0.22
	TG	116.6 ± 51.5	0.18	124.3 ± 53.8	107.5 ± 47.1	***p*** **=** **0.01**
	UA	5.1 ± 1.5	0.15	5.3 ± 1.5	4.8 ± 1.6	***p*** **=** **0.04**
	BUN	15.2 ± 6.1	−0.06	15.2 ± 6.3	15.3 ± 5.8	*p* = 0.86
	CRE	0.8 ± 0.4	0.05	0.8 ± 0.4	0.7 ± 0.2	*p* = 0.21
	Na	140.3 ± 3.2	0.21	140.8 ± 2.5	139.7 ± 3.7	***p*** **=** **0.01**
	K	4.2 ± 0.5	0.13	4.3 ± 0.5	4.1 ± 0.5	***p*** **=** **0.04**
	Cl	103.1 ± 3.6	0.20	103.7 ± 3.2	102.6 ± 4.0	***p*** **=** **0.02**
	Glucose	99.6 ± 23.3	−0.07	98.8 ± 23.3	100.5 ± 23.3	*p* = 0.58

*Variables correlated with measured MMSE score and differences between the normal (i.e., participants whose MMSE score of 24 and above) and impaired (i.e., participants whose MMSE score under 24) groups using the Student's t-test. Important variables (|r|> 0.2 or p <0.1) are highlighted in bold*.

**Figure 1:**
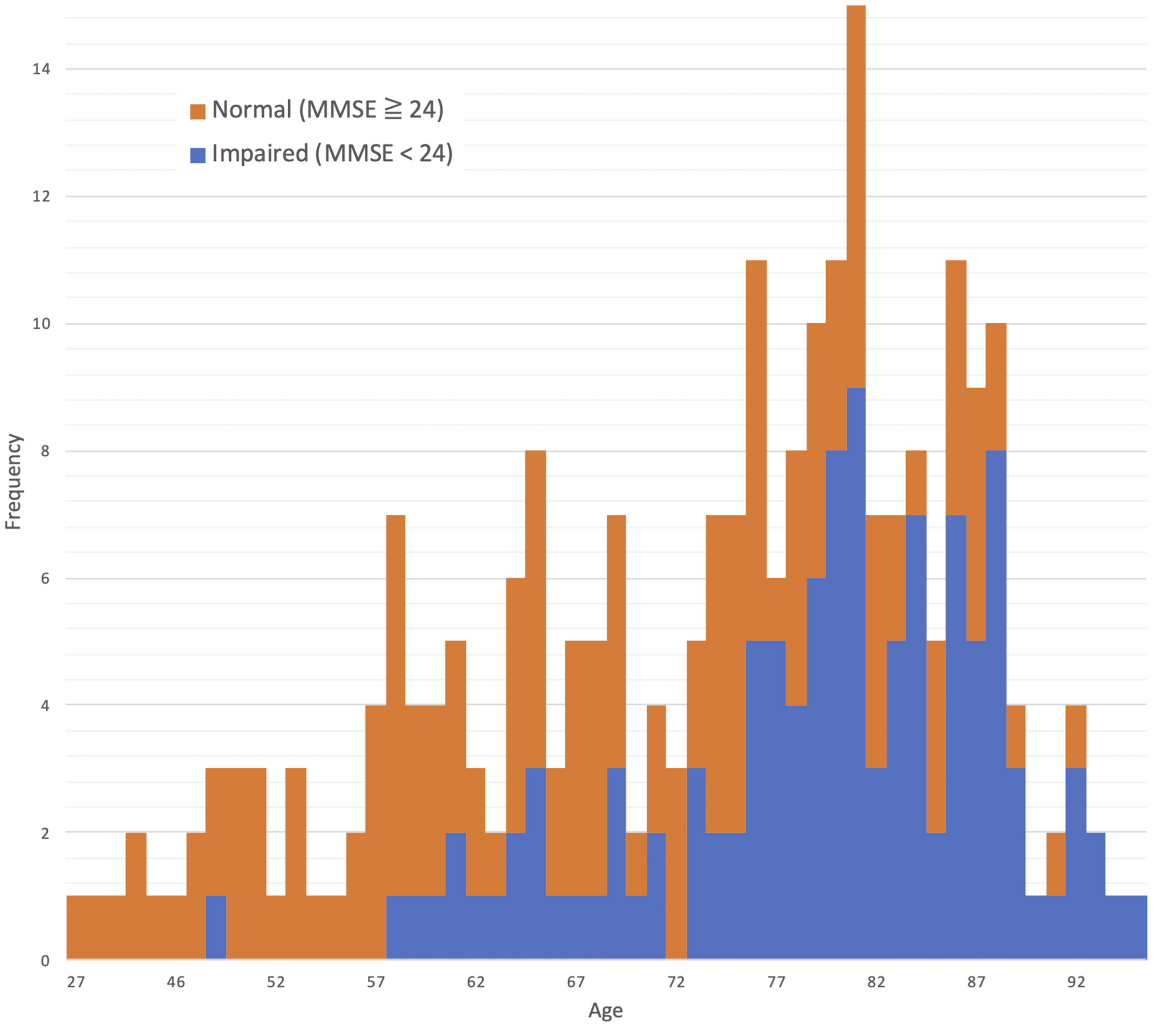
Age distribution: 73.3 ± 12.6 (mean ± SD).

**Figure 2:**
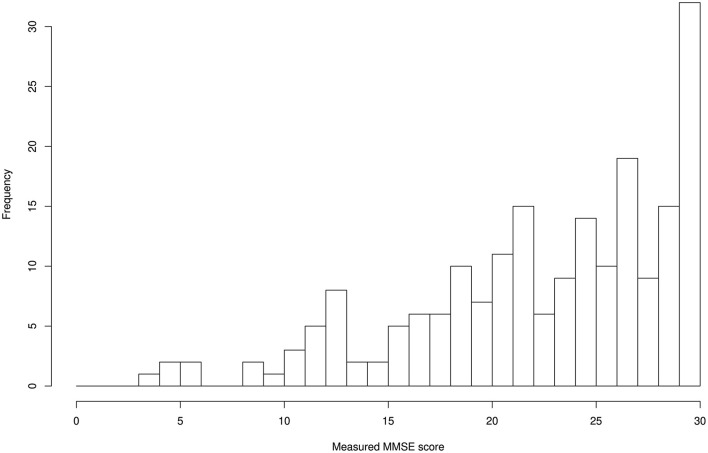
MMSE score distribution: 22.9 ± 6.1 (mean ± SD).

The distributions for age and MMSE scores are shown in Figures 1, 2, respectively. Most ages ranged from 60 to 90. The maximum score of 30 was the highest frequency for the Measured MMSE scores.

From the clinical profiles as shown in Table 3, 171 patients (68.4%) had cerebrovascular diseases (cerebral hemorrhage: 49, cerebral infarction: 100, subarachnoid hemorrhage: 22) out of the total 250 patients. 177 patients (70.8%) had at least one life-style disease including diabetes, hyperlipidemia and hypertension, while the remaining 73 patients were not suffering from life-style diseases.

**Table 3:** Clinical profiles of patients.

**Lifestyle-related diseases**
	**HT**	**DM**	**HL**	**HT**	**HT**	**HT**	**HL**	**HT**	**HT**	**HT**	**HT**	**None**	**Total**
				**DM**	**HL**	**G**	**G**	**HL**	**HL**	**DM**	**HL**		
								**DM**	**DM**	**G**	**G**		
									**G**				
CH	18	1	0	6	6	0	0	6	1	0	0	11	49
SAH	9	0	4	1	3	0	0	3	0	0	0	2	22
CI	16	3	7	12	10	2	2	14	1	1	5	27	100
HI	2	0	0	0	0	0	0	0	0	0	0	3	5
BF	16	1	0	4	4	0	0	2	0	0	0	12	39
Others	10	0	0	4	0	0	0	1	0	1	1	18	35
Total	71	5	11	27	23	2	2	26	2	2	6	73	250

*HT, hypertension; DM, diabetes mellitus; HL, hyperlipidemia; G, gout; CH, cerebral hemorrhage; SAH, subarachnoid hemorrhage; CI, cerebral infarction; HI, head injury; BF, born fracture*.

### Machine Learning Models for Data Analysis

We employed LR, RF, and neural network models to estimate MMSE scores by age, TNIRS parameters, and blood test data. LR was first investigated as the baseline model to compare prediction accuracy with the results of other learning models. LR and RF were modeled using the scikit-learn package in Python 3, whereas DNNs were performed on the Tensorflow 2 platform (13) for the data analysis. A repeated five-fold cross validation was used to thoroughly compare the results of LR, RF and DNN, i.e., 10 times of five-fold cross validation were performed for each model using the random seeds from the number of 100–109 in serial.

#### Linear Regression Model

Explainable and reproducible results are important for the first step in the analysis, and a variety of linear regression has been used for the study of Alzheimer's disease. The LR introduced in this study is the ordinary least-squares regression, which can be formulated as a set of *n* equations of the form *y*_*i*_ = *b*_0_+*b*_1_*x*_*i*1_+*b*_2_*x*_*i*2_+…+*b*_*k*_*x*_*ik*_+ε_*i*_, where *y*_*i*_ is the response for the number *i* in the sample data , *x*_*ij*_ is the predictor value, *b*_*j*_ is the coefficient, and ε_*i*_ represents an error term.

#### Random Forests

RF is a basic ensemble algorithm that combines a number of classification or regression trees and is based on the bagging technique. The RF algorithm is a powerful model as the ability to handle highly non-linearly correlated data, robustness to noise, tuning simplicity. Importantly, the applicability of RF for the prediction of Alzheimer's disease from neuroimaging data has recently been more acknowledged as one of the methods to achieve higher prediction accuracy (14). The RF classifier requires only a few hyper-parameters for tuning the performance. In the present study, we used the module *RandomForestRegressor* with the settings of the number of estimators (n_estimators: 100) and the maximum depth (max_depth: 8) in the scikit-learn package.

#### Neural Networks

The DNN for regression was modeled in the Tensorflow 2 as a multi-layer, feedforward neural network. It is noteworthy that an activation function called the scaled exponential linear unit (SELU) was chosen with the ADAM optimizer for the best accuracy in this problem domain. The combination of batch normalization, dropout, and L2 regularization as the regularization algorithms were applied during the training phase to avoid overfitting and acquire stable DNN models.

**Figure 3:**
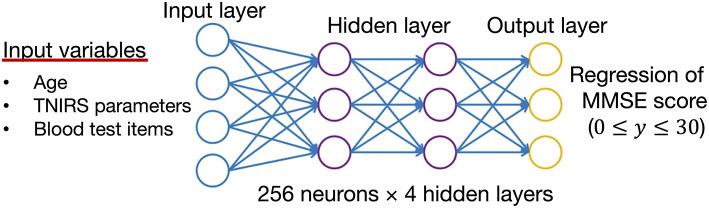
Structure of the deep neural network for data analysis. The input vectors include age, blood test data, and TNIRS data. The output vector is regressed to estimate the MMSE score. The hidden layer contains no backward connections from downstream layers.

The weighted combination $\alpha =\overset{n}{\underset{i=1}{\sum }}w_{i}x_{i}+b$ aggregates input signals *x*_*i*_ in each layer to activate an output signal *f*(α) to the connected neuron in the next layer. The DNN in this study included 35 neurons in the input layer (i.e. age, 14 variables in the TNIRS parameters, and 23 variables in the blood test items; Figure 3).

Each hidden layer was placed with 256 neurons, and the total number of hidden layers was four based on the results of a random search of hyper-parameters. The DNN model with 256 neurons and four hidden layers showed the best accuracy for this regression problem. Each function (*f* ) was used throughout the network, and the bias (*b*) accounted for the neuron's activation threshold. After examining the results from a random search of hyper-parameters, we chose SELU as it is a non-linear activation function with excellent characteristics to help normalize the input signals. Before applying SELU to each hidden layer, the algorithms for batch normalization and 10% of the dropout rate were performed for the input signals.

The output signals (*f*(α)) in each layer were determined by using a weighted combination of the input signals *x*_*i*_ from upstream of the DNN. In the output layer, a loss function, *L*(*W, B* | *j*), was measured by using the mean square error between the estimated value and the actual MMSE score. The learning process updated the weights (*W*) and biases (*B*) until the loss function, *L*(*W, B* | *j*), was minimized. Note that *W* is the collection {_*W*_*i*_}1:*N*−1_, where *W*_*i*_ denotes the weight matrix connecting layers *i* and *i*+1 for a network of *N* layers. Similarly, *B* is the collection {_*b*_*i*_}1:*N*−1_, where *b*_*i*_ denotes the column vector of biases for layer *i*+ 1.

## Results

We first evaluated correlations between the measured parameters and cognitive functions evaluated by the MMSE to discuss how prediction accuracy of the MMSE score could be improved. Then, both linear regression and DNN regression were performed. Predicted MMSE scores with substantial residual error were reviewed by referring to the participant's diagnosis results.

### Correlation Analysis

**Figure 4:**
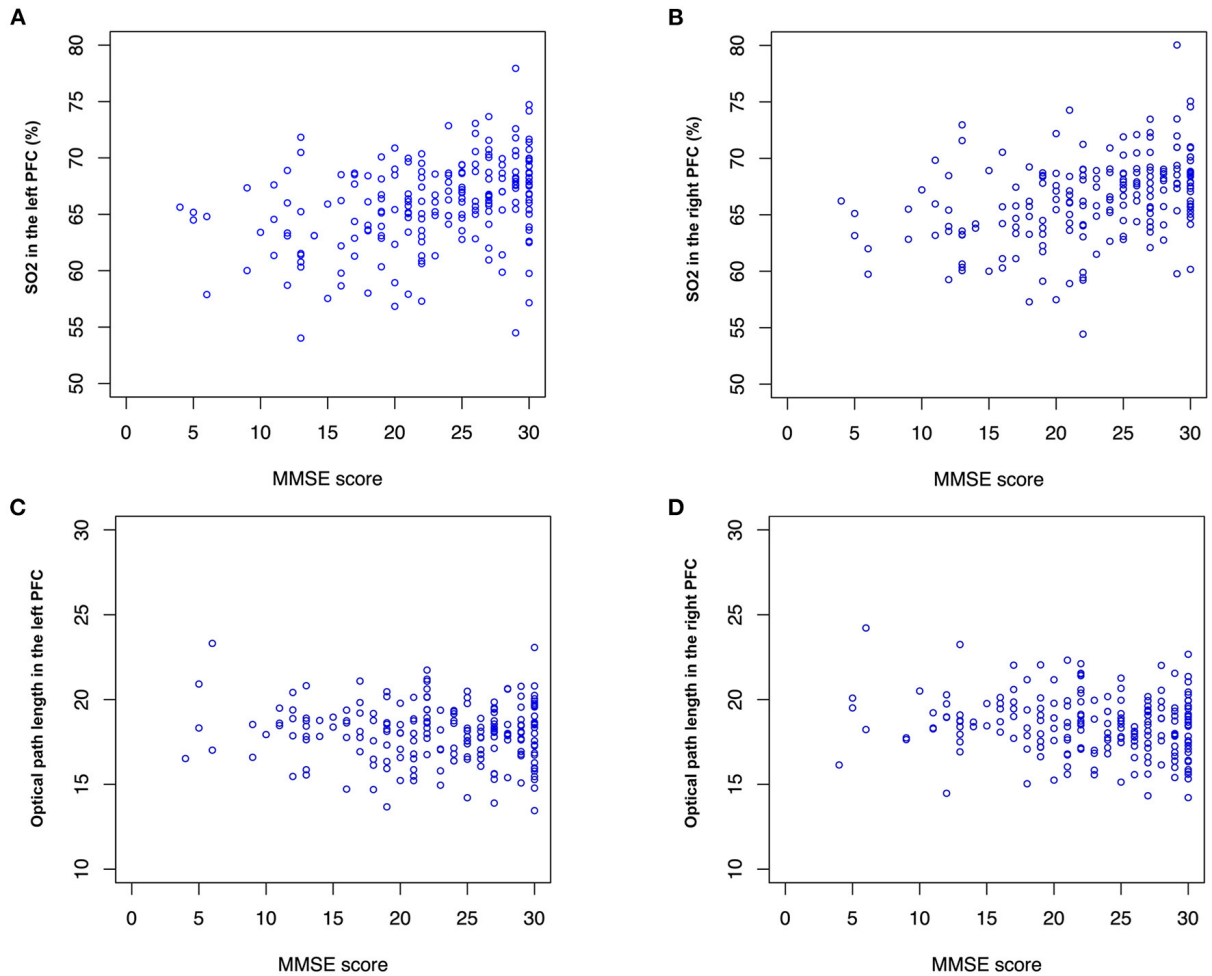
SO_2_ and optical path length (OP). MMSE score is correlated with **(A)** SO2 in the left and right PFC (*r* = 0.30, *p* <0.01) and **(B)** right PFC (*r* = 0.30, *p* <0.01). No significant correlation between MMSE score and OP is found, however, mean values of OPs in **(C)** the left PFC (mean: 17.9 and 18.0, *p* <0.1) and **(D)** the right PFC (mean: 18.2 and 18.6, *p* <0.1) are different between the normal and impaired groups.

Correlation analysis showed a significant negative correlation between the age of the participants and the MMSE scores (*r* = −0.42, *p* <0.01). Age was the most important variable because the baseline concentration of SO_2_ and blood counts changed with aging. The baseline concentration of SO_2_ in the PFC measured by TNIRS varied between participants; however, we found a significant positive correlation between MMSE and SO_2_ in the left PFC (*r* = 0.30, *p* <0.01) and the right PFC (*r* = 0.30, *p* <0.01) as shown in Figure 4. Hence, in addition to age, the baseline concentrations of SO_2_ in the left and right PFCs are useful for estimating the MMSE score using the TNIRS.

Interestingly, optical path lengths for light (791, 836 nm) transmitted through tissue in the right PFC were significantly different between the Normal and Impaired groups (*p* <0.05). Optical path lengths were inversely proportional to an MMSE score higher than 15. Note that optical path lengths (OPs) in the left PFC and the right PFC in Figure 4 were measured using the LEDs of 836 nm wavelength.

### Comparison Between Two Types of Deep Neural Networks

**Figure 5:**
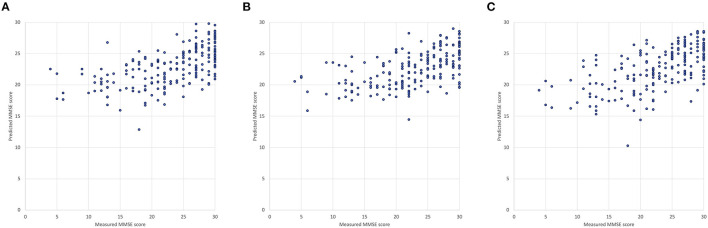
Measured and predicted scores: **(A)** DNN1 (input variables: age and 23 blood test items), **(B)** DNN2 (input variables: age and 14 TNIRS parameters) and **(C)** DNN3 (input variables: age, 14 of the TNIRS parameters and 23 blood test items).

We first compared two types of DNNs using the repeated five-fold cross validation. The sample size of training data was 202 by removing the instances containing any missing value from the data of the 250 participants. Both DNNs with 4 hidden layers were modeled with the same hyper-parameters described in Section Machine Learning Models for Data Analysis. DNN2 exhibited slightly better accuracy as MAE: 3.97 ± 0.07 (mean ± SD) and MAPE: 26.0 ± 0.3 % (mean ± SD) compared with DNN1 as MAE: 4.11 ± 0.07 (mean ± SD) and MAPE: 26.3 ± 0.4 (mean ± SD) as shown in Figures 5A,B. It is noteworthy that DNN2, which uses 14 of the TNIRS parameters, produced the predicted MMSE scores with a smaller variance; however, these prediction results are biased as the predicted MMSE scores of the participants with the measured MMSE score under 20 are mostly higher than the actual MMSE score.

### Result From the Deep Neural Network Trained With Both Time-Resolved Near-Infrared Spectroscopy and Blood Test Data

By applying both TNIRS and blood test data, we confirmed that the third DNN (DNN3) generally improves prediction accuracy (MAE: 3.91 ± 0.07, MAPE: 25.3 ± 0.4%), which was approximately 5% better than the result of LR and 3% better than that of RF as discussed in Section Comparison Between Two Types of Deep Neural Networks. In Figure 5C, there is one outlier (measured MMSE score: 18, predicted MMSE score: 10.3) because of the extraordinary low values of 58.0% in *SO2* in the left PFC (mean: 66.6±4.0%) and 84 mEq/L in *Cl* (mean: 103.1±3.6 mEq/L), where *Age, SO2* in the left PFC, and *Cl* are the most weighted variables in DNN3. The corresponding participant suffered from both subjective symptoms of cerebral hemorrhage and congestive heart failure.

**Figure 6:**
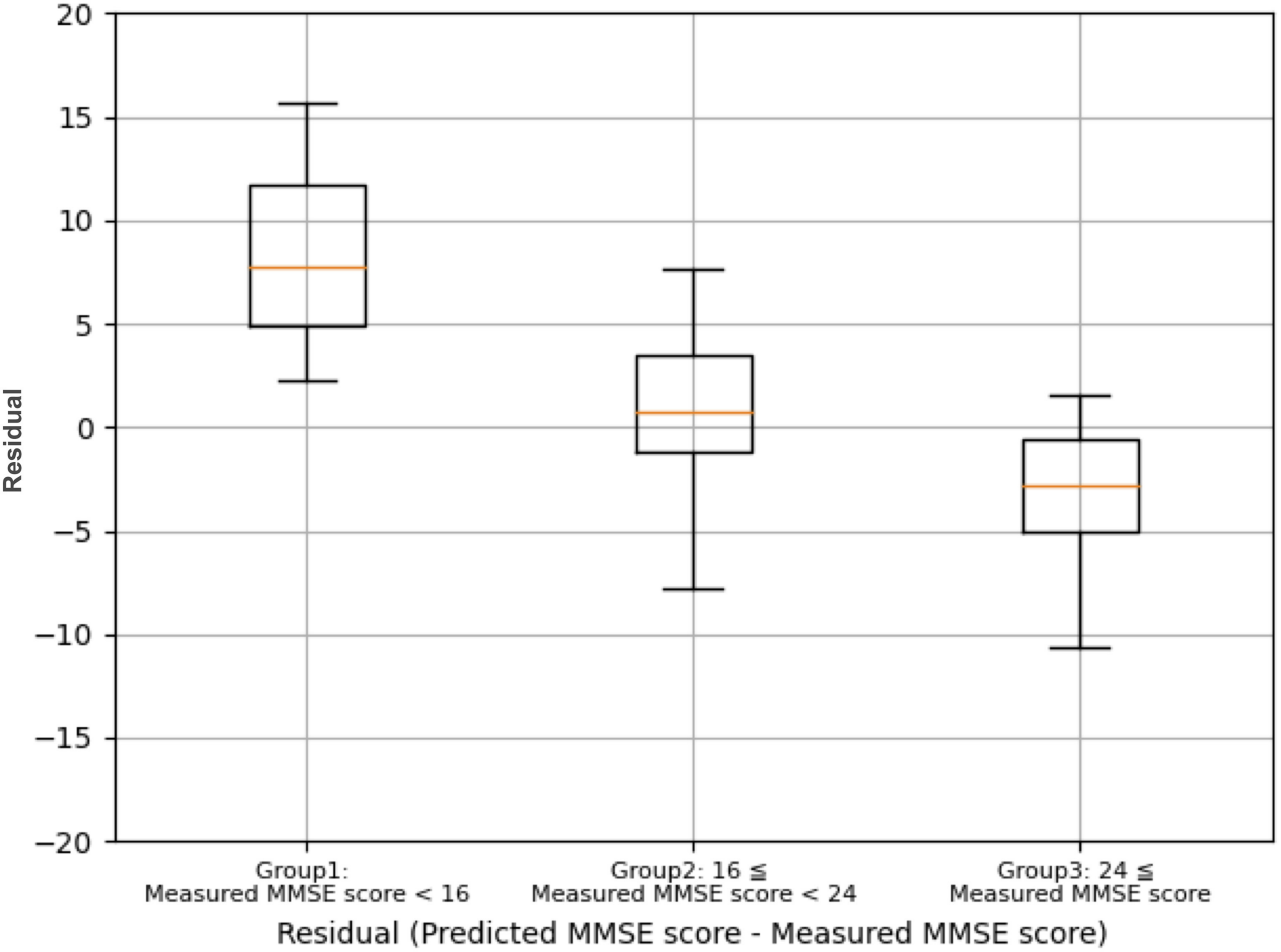
Comparison of the residual errors between group 1 (measured MMSE score <16), group 2 (measured MMSE score 16–23), and Group 3 (measured MMSE score ≥ 24).

A comparison of the residual errors between Group 1 (Measured MMSE score <16), Group 2 (Measured MMSE score 16–23), and Group 3 (Measured MMSE score >24) showed that the mean residual errors were statistically different between the three groups by one-way ANOVA (*F*_(2, 199)_ = 142.19, *p* <0.001), as shown in Figure 6. The prediction results are optimistic than the measured MMSE scores, especially for the measured MMSE score of <16.

By applying the cut-off values of ≥24 for normal and <24 for risk of cognitive impairment, as shown in Table 4, DNN3 has the prediction accuracy with 88.7% sensitivity and 100% specificity. Note that the sample size of normal participants is 69, and it is half of the ones with risk of cognitive impairment as 133.

**Table 4:** Prediction accuracy of the DNN3 in the two-class classification of MMSE (*N* = 202, five-fold cross validation).

**Ground truth MMSE**	**Predicted MMSE**		**Accuracy**
	**≥24**	** <24**	
≥ 24	69	0	Specificity: 100%
<24	15	118	Sensitivity: 88.7%

### Comparison

From the comparison of results between LR, RM, and DNNs presented in Table 5, using a repeated five-fold cross validation, we confirmed that the DNNs improved prediction accuracy compared with LR and RM. In all three versions of the DNN, the values for MAE were significantly lower (p <0.001) than the results for LR and RM. In particular, DNN3 generally improved MAPE by 5% compared to the LR.

**Table 5:** Comparison of prediction results (mean values from 10 trials of five-fold cross validation).

**Selected variables and metrics**		**Linear regression**	**Random forest**	**DNN**	**Difference between results of DNN and the others**
Blood test items (23 variables)	MAE	4.73 ± 0.17	4.56 ± 0.06	4.11 ± 0.07	*p* <0.001
	MAPE	29.1 ± 0.8 (%)	29.6 ± 0.3 (%)	**26.3** **±0.4 (%)**	
TNIRS parameters (14 variables)	MAE	4.69 ± 0.31	4.26 ± 0.09	3.97 ± 0.07	*p* <0.001
	MAPE	29.2 ± 1.1 (%)	27.7 ± 0.6 (%)	**26.0** **±0.3 (%)**	
Both input variables of blood test and TNIRS (23 + 14 variables)	MAE	5.11 ± 0.34	4.36 ± 0.09	3.91 ± 0.07	*p* <0.001
	MAPE	30.2 ± 1.3 (%)	28.3 ± 0.4 (%)	**25.3** **±0.4 (%)**	

*General improvement in MAPE was found only in DNNs (Bold)*.

Adding both input variables of blood test and TNIRS into the LR and RF did not lead to improvement in MAE. Because the number of variables becomes 38 whereas the training sample size is 200 during the repeated five-fold cross validation, these models may suffer from the curse of dimensionality (i.e., exponentially increasing sparsity to find the optimal solutions) (15). In contrast, DNN3 showed the lowest MAE even when input variables were added to this regression problem.

## Discussion

We examined the following points regarding the method of estimating cognitive function based on TNIRS data and blood test data: (1) differences in prediction accuracy between applying TNIRS and blood test data, (2) the effect of combination of TNIRS and blood test data on the prediction accuracy, and (3) differences in prediction accuracy for cognitive function between LR, RF and DNN models. We discuss each point below.

### Differences in Prediction Accuracy Between Applying TNIRS and Blood Test Data

We evaluated the prediction accuracy of cognitive function by MAE and MAPE to compare the differences between the results of TNIRS and blood test data. We found that prediction using TNIRS data exhibited lower MAE and MAPE compared with those using blood test data, however, the difference in MAPE between TNIRS and blood test data was only 0.3%. In the present study, the TNIRS probes were set on the forehead, thus measuring regional cerebral blood flow and oxygen metabolism in the PFC, which plays an important role in cognitive function (16). We reported that TNIRS parameters correlated with cognitive function expressed by MMSE in aged people (17). In contrast, blood test data reflect systemic metabolic dysfunction which affects cognitive function including malnutrition (18), anemia (19), lipid metabolism (20), purine metabolism (18), and renal function impairment (21). That is, TNIRS directly reflects cognitive function, while blood test data indirectly reflect it.

One of the DNNs (DNN3) exhibited the prediction accuracy (MAE: 3.91 ± 0.07, MAPE: 25.3 ± 0.4%), which was 88.7% sensitivity and 100% specificity by applying the cut-off values of ≥ 24 for normal and <24 for risk of cognitive impairment. On the other hand, the studies of cerebrospinal fluid (CSF) biomarkers for AD demonstrated that the accuracy and specificity were between approximately 70 and 90% (22, 23). Compared with these results, the diagnostic accuracy of our methods is similar or even higher. It can be regarded as acceptable for real clinical applications, however, the DNNs in this study were just modeled using the training data of 202 patients and random search of hyper-parameters through the repeated five-fold cross validation, aiming at the comparison with the other regression models rather than improvement in prediction accuracy. Estimation of external validity in the prediction accuracy thus should be further studied by testing various data with the conditions of different populations including healthy people. In addition, further studies are necessary to clarify the sex difference in dementia. It has often been reported that women have a higher frequency of dementia than men (24). As shown in Table 1, we observed no statistical difference between the two groups in this study, and it did not contribute to the prediction accuracy.

### The Effect of Combination of TNIRS and Blood Test Data on the Prediction Accuracy

Then, we evaluated changes in prediction accuracy when both TNIRS data and blood test data were added to the input layer in the learning models. We found that when LR was used to predict cognitive function, the combination of TNIRS and blood test data as inputs resulted in lower prediction accuracy than inputting TNIRS and blood test data separately. This phenomenon is consistent with the curse of dimensionality in linear models; that is, if the number of data dimensions becomes too large, the number of combinations that can be expressed by the data increases rapidly, and as a result, sufficient learning results cannot be obtained with finite sample data (15). With SELU activation function, dropout, and batch normalization algorithms on each hidden layer, the DNNs were able to extracted non-linear features from variables with noise and showed the highest accuracy in MAE even with both input variables of blood test and TNIRS data. Nonetheless, for the DNN models, adding TNIRS data to the blood test data of the input layer only improved MAPE by 1.0% compared to the use of blood test data alone. These results suggest that, when predicting cognitive function with DNN, adding TNIRS data to blood test data has no major effect on improving the prediction accuracy. On the other hand, the use of the blood test data alone in DNN1 exhibited the prediction accuracy with 81.8% sensitivity and 91.3% specificity, and it is rather suitable for rapid mass-screening by considering the availability of blood test data.

### Differences in Prediction Accuracy Between LR, RF and DNN Models

We also compared the prediction accuracy of cognitive function between LR, RF, and DNN. The MAE and MAPE from the results of DNN were the smallest when both TNIRS data and blood test data were added, while LR showed the worst accuracy (MAE: 5.11 ± 0.34, MAPE: 30.2 ± 1.3%) by adding the both data. In particular, there is a strong correlation between SO_2_ in the left PFC and right PFC (*r* = 0.79, *p* <0.01), and SO_2_ in the left and right PFCs are relatively weighted in LR. For the non-linear models, RF and DNN, prediction accuracies improved when both TNIRS data and blood test data were added. MMSE score shown in Figure 3 is undoubtedly the non-linear response, not only because the participants with the MMSE score under 16 were mostly hard to complete the test itself due to their comorbidities, but also the maximum MMSE score is 30 whereas healthy people can often achieve the score. RF also has the nature of feature selection as an ensemble of unpruned regression trees, induced from bootstrap samples in the training data, using random feature selection for the tree induction process (25). This random feature selection can prevent over-fitting to some degree. Nonetheless, there was a lot of noise in the training data of the 250 participants, and RF was not able to handle this, especially in cases where only data with noise were subsampled. From the blood test data, ~20 variables, with the exception of A/G, PLT, and Cl, had <5% variable importance. From the TNIRS data, most variables also had <5% variable importance, with the exception of SO_2_ in the left and right PFCs. Most variables in RF thus held lower weights, which were the same as the noise level.

Interestingly, the prediction accuracy for cognitive function differed depending on the MMSE score (Figure 6). The underlying mechanism is not yet clear; however, it should be considered that individuals with low MMSE scores (MMSE score <16) may suffer from more brain neuronal death compared to individuals with high MMSE scores (MMSE score >24). The TNIRS and basic blood test data in the present study reflect CBF/oxygen metabolism in the PFC and systemic metabolic disorders associated with the risk of cognitive dysfunction, respectively; however, both methods cannot detect brain neuronal cell death directly. Therefore, the DNN-based prediction for cognitive function may underestimate cognitive impairment.

### Clinical Application of the DNN-Based Prediction for Cognitive Impairment

The DNN-based prediction method of cognitive impairment seems to be superior to the existing dementia screening test such as MMSE for the following reasons. First, in contrast to the MMSE which is subjective test, the present method is objective test. Second, it is difficult to administer the MMSE to individuals with disorders such as visual and hearing impairments. In contrast, the DNN-based method evaluates cognitive function in a short time. Finally, due to these advantages, this method can perform mass screening tests of cognitive impairment for a large number of subjects in a short time.

Taking advantage of the above advantages, we are applying the DNN-based prediction method to screening tests for dementia; details were described in the previous study (4). Briefly, the test is optionally performed in general health examination. Those classified as Class A (predicted MMSE scores 28 ≤ , ≤ 30; normal) will receive general life guidance, while those assessed as Class B (predicted MMSE scores (24 ≤ , <28; suspected MCI) or Class C (predicted MMSE scores <24; suspected dementia) will be advised to visit the outpatient clinic for further consultation. In the outpatient clinic, if their cognitive function is not impaired or MCI, their systemic metabolic disorders that are risk factors for dementia will be treated by a general practitioner. If there is an apparent cognitive impairment, MRI and other imaging modalities including TNIRS will be performed. Patients diagnosed with dementia will be treated by a dementia specialist.

### Limitations of the Present Study

Finally, the limitations of the present study should be discussed. First, we validated the prediction accuracy of the DNN models. The accuracy of the DNN model of blood test data was further validated in a new test group of patients after the training process of the DNN model was done in the previous study, however, in the present study the accuracy of the DNN model of TNIRS was validated only by repeated five-fold cross validation using the training data of 202 patients, due to less availability of measured data of TNIRS parameters compared to the blood test data. Second, most of the patients for training of the DNN models received medical treatments for their systemic metabolic disorders and cerebrovascular diseases at the time of this study. These treatments might affect the training of the DNN models of blood test data and TNIRS. Third, the DNN model of blood test data might miss cognitive impairment in the patients whose cause is limited to the brain. Indeed, the DNN model of blood test data missed cognitive impairment in the patients with chronic cerebrovascular diseases who exhibited normal blood test data. The use of TNIRS may be useful to avoid such misdiagnosis using the DNN model of blood test data (6). In order to establish the DNN modes as a screening test for dementia, further studies are needed to resolve these limitations.

## Conclusion

The present study demonstrated that the DNN allowed for the prediction of cognitive function expressed by MMSE scores with high accuracy based on TNIRS and/or blood test data. We found that (1) the DNN-based prediction using TNIRS data exhibited lower MAE and MAPE compared with those using blood test data, (2) the difference in MAPE between TNIRS and blood test data was only 0.3%, (3) adding TNIRS data to the blood test data of the input layer only improved MAPE by 1.0% compared to the use of blood test data alone, whereas the use of the blood test data alone exhibited the prediction accuracy with 81.8% sensitivity and 91.3% specificity (*N* = 202, repeated five-fold cross validation). The DNN model using blood test data seems to be more suitable for mass screening of cognitive impairment at present.

## Data Availability Statement

The raw data supporting the conclusions of this article will be made available by the authors, without undue reservation.

## Ethics Statement

The studies involving human participants were reviewed and approved by Human Subjects Committee of the Rehabilitation Hospital, Southern Touhoku Kasuga Rehabilitation Hospital (Sukagawa City, Japan). The patients/participants provided their written informed consent to participate in this study.

## Author Contributions

KO is the first author of this manuscript. Data analysis using linear model and deep neural networks were conducted and discussed with the co-author. KO provided an idea of deep neural network model for this study. Then, KS advised the KO with his leadership and contributed the analysis of the experimental data. All authors approved the final version of the manuscript and agreed to be accountable for all aspects of the work by ensuring that questions related to the accuracy and integrity of any part of the work are appropriately investigated and resolved.

## Conflict of Interest

The authors declare that the research was conducted in the absence of any commercial or financial relationships that could be construed as a potential conflict of interest.

## Publisher's Note

All claims expressed in this article are solely those of the authors and do not necessarily represent those of their affiliated organizations, or those of the publisher, the editors and the reviewers. Any product that may be evaluated in this article, or claim that may be made by its manufacturer, is not guaranteed or endorsed by the publisher.
